# Scoop pen sub-study-a ‘trial within a trial’ of enclosing a pen in questionnaire mailings to increase response rate

**DOI:** 10.1186/1745-6215-16-S2-O11

**Published:** 2015-11-16

**Authors:** Caroline Fairhurst, Kerry Bell, Laura Clark, Natasha Mitchell, Elizabeth Lenaghan, Jeanette Blacklock, Lee Shepstone, David Torgerson

**Affiliations:** 1 York Trials Unit, York, UK; 2 Norwich Medical School, Norwich, UK

## Background

High response rates to postal questionnaires are essential to maximise trial retention and statistical power, and thus ensure high quality and valid results. Monetary incentives are often effective at improving response rates but are, by nature, costly. Enclosing a pen with postal questionnaires has the potential to be a cheap and effective alternative.

## Aim

To assess the effectiveness of including a pen in postal questionnaires on response rate, time to response, completeness of response and whether a reminder notice is required.

## Methods

A two-arm RCT embedded within the SCOOP trial. 3,826 women, aged between 70 -85 years, were randomised to receive a pen with their 60-month follow-up questionnaire and 3,829 to receive the questionnaire alone. The results were combined with those of a previous embedded RCT in a meta-analysis.

## Results

Of the 3,789 participants sent a 60-month questionnaire in the pen group, 3,500 (92.4%) returned it compared with 3,462/3,793 (91.3%) in the control group (OR 1.16, 95% CI 0.98 to 1.37, p=0.08). There was evidence of a reduction of the number of reminders required (p=0.02) and the time taken to respond (p=0.01) in the pen group. Some difference was found in the completeness of the primary outcome (p=0.05). The pooled OR of response rates from the meta-analysis was 1.21 (95% CI 1.05 to 1.39, p=0.01).

## Conclusions

The inclusion of a pen with postal questionnaires does potentially have a positive impact on RCT attrition and response rates, and on reducing the time to response and the numbers of reminders required. We estimate the cost per retained participant at £32, compared to £450 had we observed the same effect by sending a small, unconditional incentive of £5 with the questionnaire.

